# Technical Progress in Single-Incision Laparoscopic Cholecystectomy in Our Initial Experience

**DOI:** 10.1155/2011/972647

**Published:** 2011-04-26

**Authors:** Tomohiko Adachi, Tatsuya Okamoto, Shinichiro Ono, Takashi Kanematsu, Tamotsu Kuroki

**Affiliations:** Department of Surgery, Graduate School of Biomedical Sciences, Nagasaki University, 1-7-1 Sakamoto, Nagasaki 852-8501, Japan

## Abstract

Single-incision laparoscopic cholecystectomy (SILC) has rapidly spread throughout the world because of its low invasiveness and because it is a scarless procedure. Various surgical methods of performing SILC are present in each institute; however, it is necessary to develop a standardized procedure that we can perform safely, such as the conventional 4-port laparoscopic cholecystectomy (LC). The SILC experiment in our institute was started by use of the commercial SILS Port and changed from a 3-port method via an umbilicus to a 2-port method to improve some problems. Although none of the conversions to conventional 4-port LC and also none of the complications such as bile duct injury occurred in each method, the 2-port method functioned best and was also economical. However, it is most important to adopt strict criteria and select the patients suitable for SILC to demonstrate SILC safety same as 4-port LC.

## 1. Introduction

Laparoscopic cholecystectomy (LC) was first demonstrated by Philippe Mouret in France in 1987 [1]. Since then, LC has become the standard procedure for the treatment of gallstones, cholecystitis, or gallbladder polyps. Traditionally, LC has involved four ports. Many laparoscopic techniques have been developed using this 4-port LC, and it has become possible to perform these techniques safely. Now, having established the safety of LC, our interest focused on reducing the invasiveness and scarring caused by the procedure. Cuesta et al. reported single-incision laparoscopic cholecystectomy (SILC), in which two 5 mm ports were introduced through the umbilicus, and a Kirschner wire hook was introduced through the right subcostal area to pull in an upright direction in order to visualize Calot's triangle [2]. Several surgeons have described performing SILC using three 5 mm ports from the umbilicus [3, 4]. Meanwhile, Merchant et al. also performed SILC by inserting a Gelport (Applied Medical, Rancho Santa Margarita, CA, USA) to stretch the umbilical fascia incision for easy access with instruments into the abdominal cavity [5]. Furthermore, a technique involving several transumbilical-placed ports for single-incision laparoscopic surgery was newly developed, and SILC by means of the ASC Triport (Advanced Surgical Concepts, Wicklow, Ireland) has been described successively [6–8]. On the other hand, an interesting new instrument named SPIDER (TransEnterix, Inc., Research Triangle Park, NC) for use in single-incision surgery was developed, and its use in SILC in an animal experiment has been reported [9]. As a result of these clinical studies, the use of SILC has spread rapidly. Various ports and instruments are available, and various surgical methods used in performing SILC are available in many institutions; however, it is necessary to develop an excellent procedure that can be performed safely like the conventional 4-port LC, and it is also necessary to balance safety, operativity, and economy in this new technique. 

We herein describe the experience with SILC in our institute, focusing on the technical problems and the advances made to overcome these problems.

## 2. SILS Port Procedures

**Figure 1:**
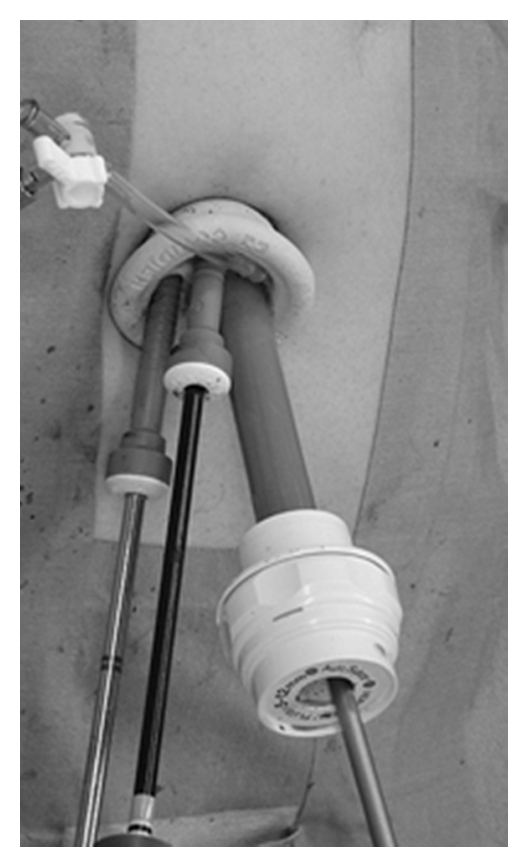
External view of SILS Port. Easy replacement of a 5 mm port with a 12 mm port is one of the advantages of this port.

In performing SILC, we first selected the SILS Port (Covidien, Inc., Norwalk, CT, USA) (Figure 1). This port was developed for use in single-incision laparoscopic surgery, and it has contributed to the global spread of SILC. The approximate operative procedures using this SILS Port are as follows. Under general anesthesia, an approximately 25 mm vertical skin incision was made through the center of the umbilicus, the peritoneal cavity was entered with the open method, and then the SILS Port was inserted. Three exclusive 5 mm ports were inserted through the SILS Port, and one 5 mm port was changeable to an exclusive 12 mm port. The pneumoperitoneum was set at 8 mm Hg, and a 5 mm flexible scope (Olympus, Tokyo, Japan) was used for the intra-abdominal visualization. A 2 mm loop-type retractor (Miniloop retractor II; Covidien) was inserted directly in the right subcostal area. After the patient was placed in the reverse Trendelenburg position and slightly rotated to the left, the fundus of the gallbladder was tightened by means of this loop-type retractor, and the gallbladder was thereafter suspended. In dissecting the gallbladder, a curved grasper, bipolar forceps, or monopolar hooks were used from the two remaining apertures. The cystic duct and artery were exposed and clipped with a 5 mm clip applier (EndoClip; Covidien) and then divided with laparoscopic scissors. The gallbladder was extracted with an endoscopic retrieval bag (Endocatch GOLD; Covidien).

Actually, SILC using the SILS Port was demonstrated to be as safe as conventional 4-port LC, and complications such as bile duct injury or uncontrolled bleeding did not occur. However, the problem areas where improvements are needed are the following: (1) the umbilical scar via the SILS Port was larger than that of conventional 4-port LC. Concretely, the umbilical scar length in the case of conventional 4-port LC was about 15 mm; however, using the SILS Port, it was approximately 25 mm, and furthermore in cases where the umbilicus bottom was shallow, the scar might be unexpectedly large. (2) Conflict between the operative instruments and the scope was inevitable, and the procedure was also inconvenient to perform because the surgeon and the assistant had to stand at the same side of the patient. Therefore, we strongly recognized the necessity to improve the new SILC technique without using the SILS Port.

## 3. Three-Port Method via Umbilical Incision

**Figure 2:**
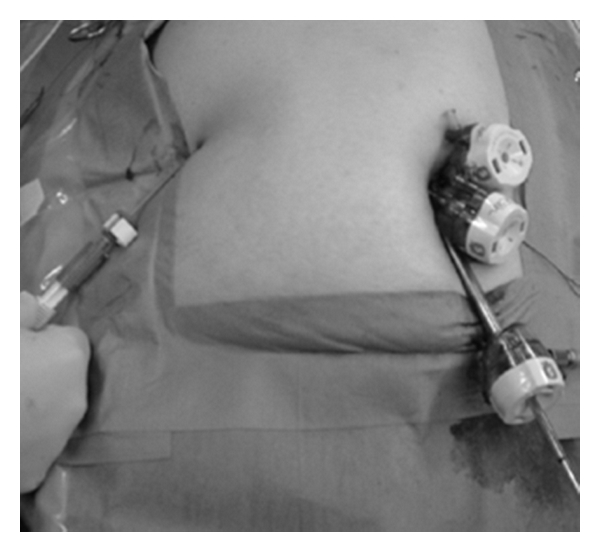
External view of 3-port LC via umbilical incision.

Next, we selected the 3-port method, which makes use of three 5 mm ports (Ethicon, Brunswick, NJ, USA) (Figure 2) via umbilical incision. The same forceps, graspers, or electrical devices were used as when using the SILS Port. This technique was able to shorten the length of the umbilical scar by approximately 5 mm in comparison to the use of the SILS Port; however, the conflicts between the operative instruments and the scope and between the surgeon and the assistant were not improved. As a result, it was found that the ideal technique for SILC would involve the insertion of only two ports via umbilical incision and would have the surgeon and the assistant located on opposite sides of the patient.

## 4. Two-Port via Umbilical Incision

**Figure 3:**
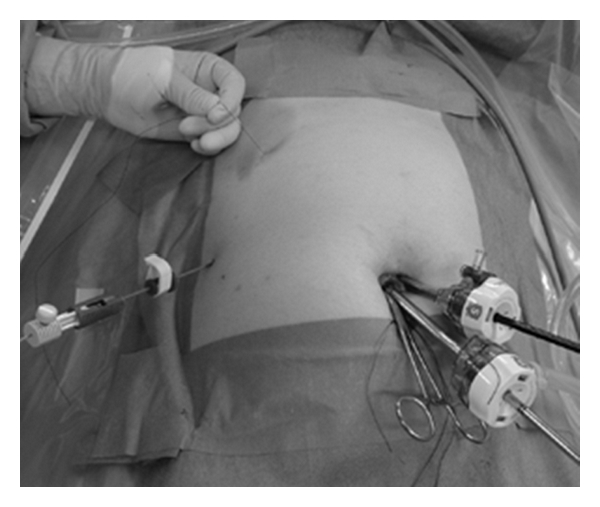
External view of 2-port LC. The surgeon operates one instrument and a 5 mm flexible scope by herself, and the assistant pulls or pushes the fine loop retractor and the nylon suture. In this photograph, the assistant pulls a nylon suture with his left hand.

**Figure 4:**
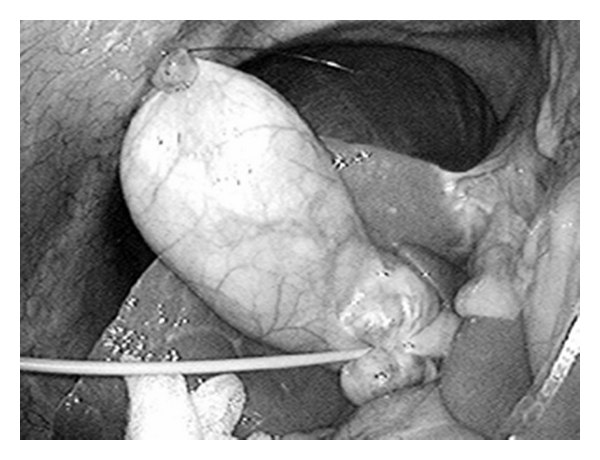
The nylon suture elevates the gallbladder and a fine loop-type retractor pulling the infundibulum presents Calot's triangle.

A 15 mm vertical skin incision was made through the center of the umbilicus. After the fascia was exposed, two 5 mm ports were introduced at separate sites, one on the left side for the 5 mm laparoscopic flexible scope and one on the right side for a forceps or grasper to dissect the gallbladder. The instruments used in this technique were the same as in the conventional 4-port LC. A 2 mm loop-type retractor was inserted from the right subcostal arch to present Calot's triangle by extending Hartman's pouch. A nylon suture with a straight needle to which a Roeder knot [10] was added to the end was inserted through a 5 mm left side port. The fundus of the gallbladder was tightened with the Roeder knot, and then the straight needle was inserted from the abdominal cavity to the right subcostal abdominal wall (Figure 3). The gallbladder was elevated by raising this nylon suture, and a good surgical field was obtained (Figure 4). The surgeon operated both one instrument and the 5 mm flexible scope by herself, and the assistant made a good surgical field such as Calot's triangle via the traction of the gallbladder using a fine loop retractor and nylon suture. This technique relieved the interference between the surgeon and the assistant and between the forceps themselves. To extract the exfoliated gallbladder, one 5 mm port was removed, and an endoscopic retrieval bag was inserted directly with an original hole, and the gallbladder was then extracted. No intraperitoneal drainage was used. The fascial defect of the umbilicus incision was repaired with approximately two stitches, and an intradermal suture was performed on the skin. The treatment of the small scar made by the 2 mm loop-type retractor and nylon suture was unnecessary. This technique represents minimally invasive surgery that combines low invasiveness and with a scarless outcome.

Another advantage of this technique is that it is inexpensive, as the instrumental cost could be reduced by approximately 170 US dollars in comparison with the conventional 4-port LC. As another advantage, when cholecystectomy by means of this 2-port technique is difficult due to severe inflammation or intraperitoneal adhesion, we could immediately shift to conventional 4-port LC using the same instruments. More specifically, the right side 5-mm port inserted via the umbilical incision would be withdrawn and reinserted via the processus xiphoideus below, and an additional 5 mm port would be introduced in the right subcostal area. A 2 mm loop-type retractor could be used to lift the gallbladder. By this technique, conventional LC can be performed. The air leak from the foramen after the 5 mm port is withdrawn is small. This simple transition is also a great advantage of our 2-port technique because it can be made in any case of cholecystitis or intraperitoneal adhesion.

## 5. Discussion

With the global expansion of the use of SILC, large series of cases have been reported in many institutes. Curcillo et al. reported in their multi-institutional 297-case series that the use of an additional port outside the umbilicus occurred in only 34 cases, and they concluded that SILC was safe and might serve as an alternative to multiport therapy with fewer scars and better cosmesis [11]. Erbella and Bunch surprisingly reported that their mean operative time was 30 min (from 22 to 75 min) in 100 consecutive SILC cases [12]. Rivas et al. reported that they had observed surgeons in training and found that experienced laparoscopic surgeons might not need to undergo a steep learning curve, and they concluded that SILC was becoming the standard procedure for most elective patients with gallbladder disease [13]. Other reports also concluded that SILC was safe [14, 15]; however, Hernandez et al. reported that biliary complication (cystic duct stump leak) occurred in one of 100 SILC cases [16], and Edwards et al. described that biliary complications occurred in 3.7% of their SILC patients (cystic duct stump leak; 1, accessory duct leak; 2) [17]. Moreover, iatrogenic combined bile duct and right hepatic artery injury during SILC has already been reported [18], and the authors recommended that surgeons should have a low threshold to add additional ports when necessary to ensure that procedures were completed safely, especially in their initial stages. As described, SILC is a useful technique; however, it is necessary to assure that the procedure is as safe as conventional 4-port LC. In our department, to secure the safety, acute cholecystitis is excluded from the indication for SILC for the present. 

Comparative studies between SILC and conventional 4-port LC regarding operating time, operative cost, complications, postoperative pain, cosmetic result, and time to return to normal activity have been performed gradually over time. Fronza et al. reported that the operating time was significantly longer in SILC, and 12% of SILC patients were readmitted within 24 hours after the operation although these readmissions were due to complications similar to those found in 4-port LC [19]. Similarly, Chang et al. concluded that there was a significant difference in operative time (SILC was approximately 1.6 times longer) and in operative cost (SILC was 1.29 times more expensive), but no difference in postoperative pain was observed [20]. However, their result that patients who underwent SILC returned to normal activity 1.8 days earlier than 4-port LC patients seems to demonstrate the usefulness of SILC. Furthermore, two randomized controlled trials (RCTs) that compared SILC with conventional 4-port LC have already been published [21, 22]. One of these trials included 70 patients, and the other included 40 patients. In a result common to both trials, the operating time in SILC was longer than that in 4-port LC, while it was found that the two methods differed in terms of the patients' post-operative pain. According to the conventional reports, the benefit of SILC has not yet become clear; therefore, well designed RCTs are needed to evaluate the corrective operative outcomes and the necessity of SILC. 

## 6. Conclusion

LC has reached an important turning point with the development of single-incision laparoscopic surgery. Further efforts and research will bring about improvements in SILC; however, it is crucial that we are able to assure that the procedure is as safe as 4-port LC. Also, especially in the early use of this procedure, we have to adopt strict criteria and select ideal patients.
